# MicroRNA-532 and microRNA-3064 inhibit cell proliferation and invasion by acting as direct regulators of human telomerase reverse transcriptase in ovarian cancer

**DOI:** 10.1371/journal.pone.0173912

**Published:** 2017-03-14

**Authors:** Lin Bai, Hui Wang, Ai-Hua Wang, Luo-Ying Zhang, Jie Bai

**Affiliations:** Department of Obstetrics and Gynecology, the First People's Hospital of Shangqiu, Shangqiu, China; Universitat des Saarlandes, GERMANY

## Abstract

Human telomerase reverse transcriptase (hTERT) plays a crucial role in ovarian cancer (OC) progression. However, the mechanisms underlying hTERT upregulation in OC, and the specific microRNAs (miRNAs) involved in the regulation of hTERT in OC cells, remains unclear. We performed a bioinformatics search to identify potential miRNAs that bind to the 3'-untranslated region (3'-UTR) region of the hTERT mRNA. We examined the expression levels of miR-532/miR-3064 in OC tissues and normal ovarian tissues, and analyzed the correlation between miRNA expression and OC patient outcomes. The impacts of miR-532/miR-3064 on hTERT expression were evaluated by western blot analysis and hTERT 3'-UTR reporter assays. We investigated the effects of miR-532/miR-3064 on proliferation and invasion in OC cells. We found that miR-532 and miR-3064 are down-regulated in OC specimens. We observed a significant association between reduced miR-532/miR-3064 expression and poorer survival of patients with OC. We confirmed that in OC cells, these two miRNAs downregulate hTERT levels by directly targeting its 3'-UTR region, and inhibited proliferation, EMT and invasion of OC cells. In addition, the overexpression of the hTERT cDNA lacking the 3'-UTR partially restored miR-532/miR-3064-inhibited OC cell proliferation and invasion. The silencing of hTERT by siRNA oligonucleotides abolished these malignant features, and phenocopied the effects of miR-532/miR-3064 overexpression. Furthermore, overexpression of miR-532/miR-3064 inhibits the growth of OC cells *in vivo*. Our findings demonstrate a miR-532/miR-3064-mediated mechanism responsible for hTERT upregulation in OC cells, and reveal a possibility of targeting miR-532/miR-3064 for future treatment of OC.

## Introduction

Ovarian cancer (OC) is a highly aggressive disease and represents the most lethal gynecologic tumor in the world [1, 2]. The high mortality rate of OC has been attributed to a lack of understanding of molecular mechanisms underlying OC metastasis. Recent advances have implicated numerous genetic and epigenetic alterations in the underlying biology of OC metastasis. In human tumors, telomeres are continuously elongated by human telomerase reverse transcriptase (hTERT, the catalytic subunit of the telomerase complex), which adds repeat sequences of DNA to the ends of chromosomes [3]. hTERT is widely expressed in more than 85% of human tumors including OC [4], and it has been shown to promote the epithelial-to-mesenchymal transition (EMT), invasion and proliferation of OC cells [5, 6]. The transcriptional factor Slug is a well-documented EMT initiator in different cancers [7, 8], and hTERT induces OC cell invasion by upregulating Slug expression [5, 9]. The siRNA-mediated knockdown of hTERT and targeted inhibition of telomerase activity using the telomerase inhibitor BIBR1532 decreased hTERT expression and inhibited the proliferation and invasion of tumor cells [10, 11].

Small regulatory RNAs such as microRNAs (miRNAs), have been linked to cancer development, and are reported to suppress hTERT expression in various cancer cells [12–15]. It is noteworthy that a single protein-coding gene can be regulated by several miRNAs [16]. Although a previous study has shown a direct interaction between hTERT mRNA and miR-498 in OC cells [17], the epigenetic mechanisms underlying hTERT upregulation in OC and tumor suppressor miRNAs involved in the regulation of hTERT in OC cells, are still not fully understood.

This study shows that miR-532 and miR-3064 that were down-regulated in OC tissues, can suppress the proliferation, EMT and invasion of OC cells by directly repressing hTERT expression.

## Material and methods

### Ethics statement

We obtained 60 specimens of serous OCs form the Department of Obstetrics and Gynecology, the First People's Hospital of Shangqiu (Shangqiu, China). A total of 20 normal ovarian tissues were collected as controls from patients following surgery for uterine fibroids. All subjects provided written consent. The study was conducted in accordance with the Declaration of Helsinki, and the protocol was approved by the Ethics Committee of the First People's Hospital of Shangqiu (reference number 2010–104). All tissue samples were immediately snap-frozen in liquid nitrogen. Total RNA was isolated using TRIzol reagents (Invitrogen, CA, USA).

### Cell culture, reagents and transfections

Human OC cell lines (SKOV-3 and ES-2) were purchased from the Cell Bank of the Chinese Academy of Science (Shanghai, China). Normal ovarian epithelial cells (NOEC) were obtained from Pricells Biotechnology & Medicine (Wuhan, China). All cell lines used in this study were maintained in DMEM/F12 (Invitrogen, CA, USA) supplemented with 10% fetal bovine serum (FBS, Invitrogen, CA, USA). All miRNA mimics (30 nM), miRNA inhibitors (30 nM) and siRNAs (5 nM) were obtained from GenePharma (Shanghai, China), and were transiently transfected into OC cells using Lipofectamine 2000 (Invitrogen, CA, USA) following the manufacturer’s protocol. Transfection of hTERT cDNA plasmids (OriGene, MD, USA) were also performed using Lipofectamine 2000 (Invitrogen, CA, USA).

### Real-time quantitative RT-PCR (qPCR)

The total RNA was isolated using TRIzol reagents (Invitrogen), and was reverse-transcribed into cDNA using the PrimeScript RT reagent kit (TaKaRa, Dalian, China) according to the manufacturer’s instructions. The resulting cDNAs were PCR amplified using the following gene-specific primers [5]: hTERT (forward): 5′-CGGAAGAGTGTCTGGAGCAA-3′, hTERT (reverse): 5′-GGATGAAGCGGAGTCTGGA-3′, glyceraldehyde 3-phosphate dehydrogenase (GAPDH; forward): 5′-CAATGACCCCTTCATTGACC-3′ and GAPDH (reverse): 5′-GACAAGCTTCCCGTTCTCAG-3′. For the measurement of mRNA, real-time PCRs were performed using the Takara SYBR Premix Ex Taq II (Takara, Tokyo, Japan) in a 7500 Real-Time PCR System (Applied Biosystems). The NCode miRNA qRT-PCR analysis (Invitrogen, CA, USA) was used for detecting mature miRNA levels according to the manufacturer’s instructions. Forward primers are the exact sequences of the mature miR-532 and miR-3064. The housekeeping gene GADPH and small noncoding RNA U6 were used as internal controls for mRNA and miRNA quantification, respectively. All results were represented as the fold change relative to respective controls.

### Immunoblotting

The total cell lysates were prepared 48 hours post-transfection as described [18]. Protein extracts (40 μg) were electrophoresed through 10% polyacrylamide gels and transferred to a nitrocellulose membrane. The membrane was incubated with antibodies against hTERT (1:1000, Abcam, MA, USA), Slug (1:1000, Santa Cruz), Bax (1:1000, Santa Cruz, CA, USA), E-cadherin (1:1000, Santa Cruz, CA, USA) and GAPDH (1:2000, Santa Cruz, CA, USA). Western blots were developed using ECL Detection Reagents (GE Healthcare), and signals were observed using an Odyssey Infrared Imaging system (LI-COR Biosciences, Lincoln).

### Dual luciferase reporter assay

For 3'-UTR reporter assay, cells were co-transfected with 100 ng of hTERT 3'-UTR luciferase vector (OriGene), 10 ng of Renilla report plasmid (pRL-CMV, Promega, WI, USA), together with and 30 nM miRNA mimics or miRNA inhibitors with Lipofectamine 2000 (Invitrogen, CA, USA). Then cells were lysed 24 hours later and assayed for luciferase activity using the Dual Luciferase Assay Kit (Promega, WI, USA). Predicted miR-532- or miR-3064-binding sites were mutated by RiboBio Co., Ltd. (Guangzhou, China) using the QuickChange II XL Site-Directed Mutagenesis Kit (Stratagene, CA, USA). The relative luciferase activity was calculated as a ratio of firefly luciferase activity divided by Renilla luciferase activity.

### Cell counting kit-8 assay

OC cells were seeded at a density of 5 × 10^3^ per well in 96-well plates for 24 hours, and transfected as indicated. After 72 hours, 10 μl of Cell counting kit-8 solution (Dojindo, Japan) was added to each well and incubated at 37°C for 2 hours; followed by the measurement of absorbance using an automated micro-plate reader (Corona Electric) at 450 nm.

### *In vitro* transwell invasion assay

OC cells (5 × 10^4^) were plated into upper chamber of Boyden chambers coated with Matrigel as described previously [19, 20]. Complete medium containing 10% FBS was added to the bottom chamber as a chemoattractant. The chambers were incubated for 24 hours. After incubation, the non-invading cells in the upper chamber were removed with cotton swabs. Invaded cells on the bottom surface of chamber were fixed, stained with Giemsa and counted using ImageJ software (NIH).

### Lentiviral overexpression of miR-532 and miR-3064

Lentiviral vectors (pEZX-MR03) for overexpression of miR-532/miR-3064 and the negative control lentiviral vector were purchased from Applied Biological Materials Inc. (ABM, MC, Canada), and were prepared in accordance with standard protocols. ES-2 cells were infected with lentivirus and selected using 1 μg/ml puromycin for 4 weeks, to establish stable miR-532, miR-3064 or negative control (Neg) transfectants.

### Xenograft assay

The animal protocol was approved by the First People's Hospital of Shangqiu’s Institutional Animal Care and Use Committee (IACUC, ID: 2015-10-15) and Ethics committee. BALB/c nude mice (five weeks old) were purchased from Beijing HFK Bioscience (Beijing, China) and maintained under pathogen-free conditions. For the subcutaneous tumor growth assay, 1 × 10^6^ ES-2 cells were subcutaneously injected in 0.1 ml of PBS into nude mice (n = 8 per group). After implantation for 7 days, tumor volume measurement began and was performed every 4 days, using the following formula: volume = length (mm) x width^2^ (mm^2^)/2. All the mice were sacrificed on the 26^th^ day post-injection and the xenografts tissues were collected for immunohistochemical staining analysis. The xenografts tissues were formalin-fixed/paraffin-embedded and cut into 4 μm slides. The primary antibody used was anti‑Ki-67 (1:100, Cell Signaling Technology, MA, USA). Staining was performed using the IHC detection kit (Mingrui Biotechnology, Shanghai, China).

### Statistical analysis

Statistical differences were analyzed using SPSS 17.0. 2-tailed Student’s *t*-test or Fisher's exact test was used for statistical analysis. Kaplan-Meier survival curves were plotted and the log-rank test was performed. Data are shown as mean ± SD of at least three independent experiments performed in triplicate. *P* < 0.05 were considered statistically significant.

## Results

### Low expression of miR-532/miR-3064 is associated with poor survival of patients with OC

**Fig 1:**
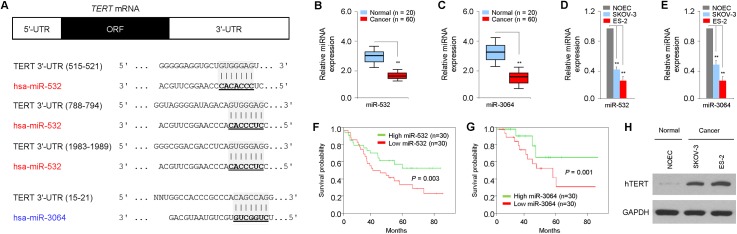
Low expression of miR-532/miR-3064 is associated with poor survival of patients with OC. (**A**) A schematic diagram explains base pairing between miR-532/3064 with the 3'-UTR sequences of hTERT; (**B, C**) qPCR analysis of miR-532 (**B**)/miR-3064 (**C**) levels in 60 OC patient samples and 20 normal ovarian tissue samples; (**D, E**) Endogenous expression of miR-532 (**D**)/miR-3064 (**E)** was determined using qPCRs in OC cell lines (SKOV-3 and ES-2) and normal ovarian epithelial NOEC cells; (**F, G**) OC patients were categorized into two groups with high (above median, n = 30) or low (below median, n = 30) miRNA expression. Kaplan-Meier survival curves for OC patients were plotted based on high or low miR-532 (**F**)/miR-3064 (**G**) expression; (**H**) Western blot analysis for the expression of hTERT or GAPDH in SKOV-3, ES-2 and NOEC cells. **: *P* < 0.01.

Using target prediction programs (TargetScan and DIANA-MicroT-CDS), we found that hTERT contains seed sequences for two putative miRNAs (miR-532 and miR-3064) in its 3'-UTR. Detailed information about these two miRNAs and their binding site sequences in the hTERT 3'-UTR are summarized in Fig 1A. Transient overexpression of miR-532 and miR-3064 resulted in significant repression of hTERT mRNA in human OC cell lines (ES-2 and SKOV-3) (data not shown). Therefore, we selected miR-532 and miR-3064 as our experimental targets.

We screened OC patient clinical tissues (n = 60) and normal ovarian epithelial tissues (n = 20) for the endogenous expression of miR-532 and miR-3064 using qPCR. We found that the expression of miR-532/miR-3064 was significantly down-regulated in OC tissues compared with normal specimens (Fig 1B and 1C). We further analyzed the expression of miR-532/miR-3064 in OC cell lines and normal ovarian epithelial NOEC cells. Our analysis revealed lower levels of both miR-532 and miR-3064 in ES-2 and SKOV-3 cells than that in NOEC cells (Fig 1D and 1E), indicating that these two miRNAs are potential tumor suppressors in OC.

To delineate the clinical significance of miR-532 or miR-3064, we determined the correlations between the levels of miR-532/miR-3064 and clinicopathological factors. 60 OC patients were divided into two groups with higher (n = 30) or lower (n = 30) expression of miR-532 or miR-3064, using the median expression values of miR-532 or miR-3064 in OC samples. More importantly, lower levels of miR-532/miR-3064 were significantly associated with advanced tumor stage, higher tumor grade and higher incidence of lymph node metastasis (S1 Table). Furthermore, we analyzed whether miR-532/miR-3064 expression levels correlate with human OC patient survival. Kaplan-Meier analysis demonstrated that reduced expression of either miR-532 or miR-3064 was significantly associated with poorer prognosis in patients with OC (Fig 1F and 1G). Moreover, high levels of hTERT were observed in ES-2 and SKOV-3 cells expressing decreased levels of miR-532/miR-3064, while a low level of hTERT was observed in NOEC cells (Fig 1H), suggesting that miR-532/miR-3064 expression inversely correlates with hTERT expression in OC cells, and the repression of these two miRNAs might be important for OC growth and/or progression.

### Identification of hTERT as a target for miR-532 and miR-3064 in OC cells

**Fig 2:**
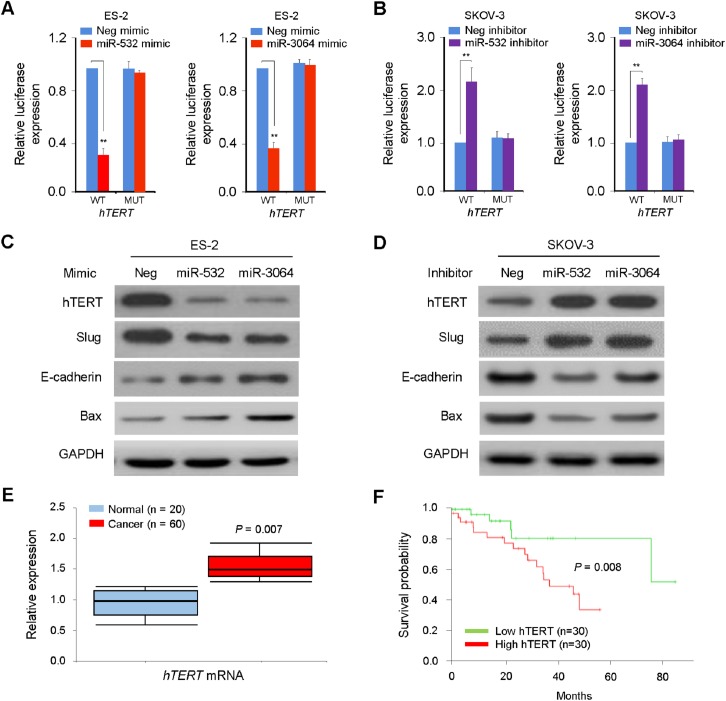
Identification of hTERT as a target for miR-532 and miR-3064 in OC cells. (**A, B**) Luciferase assay was performed in OC cells that were co-transfected with reporter vectors carrying either the wild-type (WT) version or the mutated (MUT) version of hTERT 3'-UTR, along with either miR-532/miR-3064 mimics (**A**) or miR-532/miR-3064 inhibitors (**B**). Neg mimic: negative control miRNA mimic; Neg inhibitor: negative control miRNA inhibitor; (**C, D**) Expression of hTERT, Slug, E-cadherin, Bax and GAPDH was determined using western blotting in ES-2 (**C**) or SKOV-3 (**D**) cells transfected with miR-532/miR-3064 mimics or miR-532/miR-3064 inhibitors, respectively; (**E**) The qPCR analysis of hTERT levels in OC patient samples and normal ovarian tissue samples; (**F**) Kaplan-Meier survival curves showing lower overall survival in patients with high (above median value) versus low (below median value) hTERT levels. **: *P* < 0.01.

To ascertain whether hTERT is directly targeted by miR-532/miR-3064, we performed gain-of-function experiments using ES-2 cells that express relatively low levels of miR-532/miR-3064. Our luciferase assays suggested that, the overexpression of either miR-532 or miR-3064 mimic markedly suppressed the luciferase activity of the wild-type (WT) version of hTERT 3'-UTR in ES-2 cells (Fig 2A). Conversely, the transfection of inhibitors for miR-532 or miR-3064 to another OC cell line SKOV-3 expressing relatively high levels of miR-532/miR-3064 enhanced the luciferase activity of the WT hTERT 3'-UTR (Fig 2B). To examine whether these interactions between miR-532/miR-3064 and hTERT 3'-UTR are direct, mutations targeting the predicted-binding sites of miR-532 or miR-3064 within the hTERT 3'-UTR were generated. However, the transfection with mimics or inhibitors for miR-532/miR-3064 did not significantly influence the luciferase activity of the mutated version of hTERT 3'-UTR in OC cells (Fig 2A and 2B).

Furthermore, the western blotting analysis demonstrated that the transfection with miR-532/miR-3064 mimics reduced hTERT expression (Fig 2C), while the knockdown of miR-532/miR-3064 using miRNA inhibitors increased the levels of hTERT protein (Fig 2D). Since the overexpression of hTERT induces OC cell invasion via up-regulating Slug expression [5], and the silencing of hTERT in glioma cells decreased cell proliferation by increasing the protein levels of Bax [21], we evaluated whether the modulation of hTERT levels by miR-532/miR-3064 affects the protein expressions of Slug, E-cadherin (a downstream target of Slug) and Bax in OC cells. When hTERT levels were down-regulated by the transfection of miR-532/miR-3064 mimics, Slug levels were decreased, and Bax and E-cadherin expression was induced (Fig 2C). In contrast, the silencing of miR-532/miR-3064 with miRNA inhibitors resulted in the up-regulation of Slug and down-regulation of Bax and E-cadherin (Fig 2D). Using qPCR analysis, we detected an inverse relationship between the expression of miR-532/miR-3064 and hTERT mRNA levels in OC tissues (Fig 1B and 1C; Fig 2E). Finally, we looked at the correlation between hTERT expression and OC patient survival. Higher hTERT levels in OC tissues was linked to poorer survival rate in OC patients (Fig 2F). Together, these data suggest that miR-532/miR-3064 acts directly to suppress hTERT expression in OC cells.

### miR-532/miR-3064 overexpression inhibits, whereas miR-532/miR-3064 silencing promotes proliferation and invasion in OC cells

**Fig 3:**
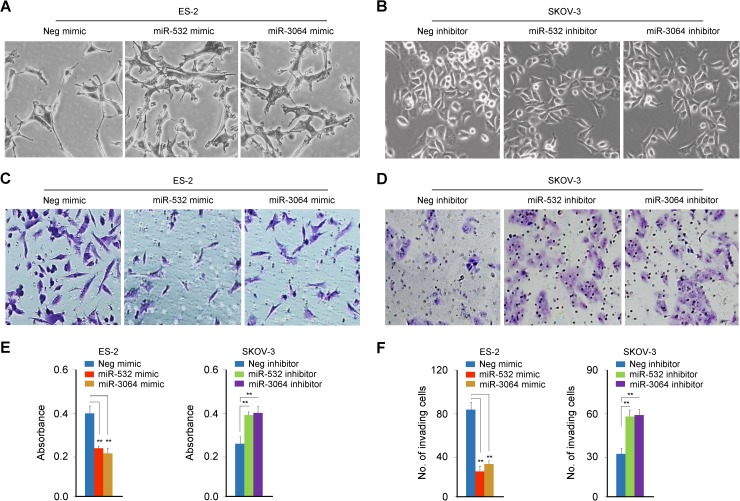
miR-532/miR-3064 overexpression inhibits, whereas miR-532/miR-3064 silencing promotes proliferation and invasion in OC cells. (**A, B**) Cell morphology of OC cells transfected with either miR-532/miR-3064 mimics (**A**) or miR-532/miR-3064 inhibitors (**B**); (**C, D**) Representative images of invaded ES-2 (**C**) and SKOV-3 (**D**) cells transfected as indicated; (**E, F**) Cell counting kit-8 assay (**E**) and transwell invasion assay (**F**) with OC cells transfected with miR-532/miR-3064 mimics or miR-532/miR-3064 inhibitors. **: *P* < 0.01.

To test the possibility that miR-532/miR-3064 suppresses OC cell proliferation and invasion, we transiently transfected ES-2 cells with miR-532/miR-3064 mimics and observed that elevating miR-532/miR-3064 levels induced a cellular phenotypic change from a fibroblastic mesenchymal morphology to a more rounded epithelial-like morphology (Fig 3A). In SKOV-3 cells, the down-regulation of miR-532/miR-3064 by miRNA inhibitors led to a scattered morphology consistent with EMT (Fig 3B). To understand the potential roles of miR-532/miR-3064 in regulating OC cell proliferation and invasion, we performed cell proliferation assay and *in vitro* cell invasion assay, and found that the ectopic expression of miR-532/miR-3064 strongly suppresses the proliferative and invasive capacity of OC cells (Fig 3C, 3E and 3F). In contrast, the silencing of miR-532 or miR-3064 stimulates OC cell proliferation and invasion (Fig 3D, 3E and 3F). These data suggest a possibility that miR-532/miR-3064 acts directly to suppress hTERT expression to suppress the EMT process and induce apoptosis in OC cells.

### hTERT is a critical mediator of miR-532/miR-3064's tumor suppressive effects in OC cells

**Fig 4:**
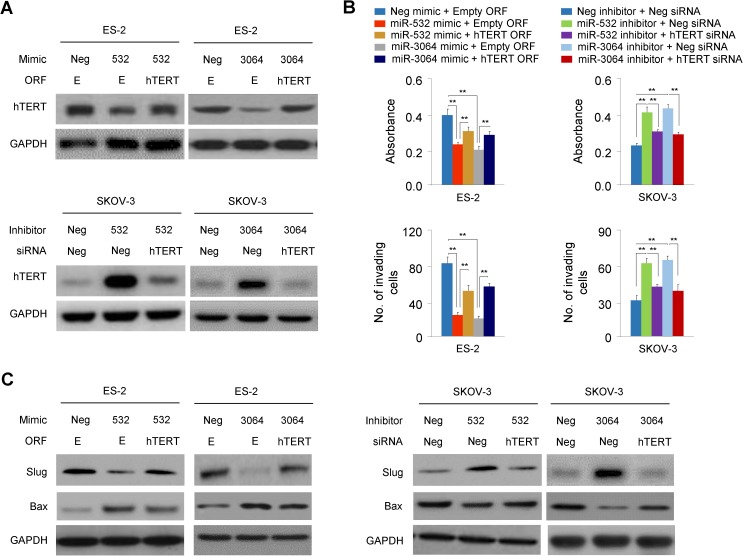
hTERT is a critical mediator of miR-532/miR-3064's tumor suppressive effects in OC cells. (**A**) Upper: western blot analysis of hTERT and GAPDH levels in ES-2 cells after transfection with miR-532/miR-3064 mimics, along with (or without) hTERT cDNA vector (ORF) lacking the 3'-UTR region. Lower: Western blot analysis was performed in SKOV-3 cells transfected with miR-532/miR-3064 inhibitors, together with (or without) hTERT siRNA; (**B**) Cell counting kit-8 assay (upper panel) and transwell invasion assay (lower panel) were performed in OC cells transfected as described above; (**C**) The protein expression of Slug and Bax in ES-2 (left panel) and SKOV-3 (right panel) cells transfected with miR-532/miR-3064 mimics (or miR-532/miR-3064 inhibitors) followed by hTERT overexpression (or hTERT knockdown). **: *P* < 0.01.

Given the oncogenic roles of hTERT in promoting OC cell proliferation and invasion [5, 6], we next determined whether hTERT serves as a critical mediator of miR-532/miR-3064's roles in cellular proliferation and invasiveness. The forced expression of the hTERT cDNA vector lacking the 3'-UTR sequence could recover hTERT protein expression in ES-2 cells transfected with miR-532/miR-3064 mimics (Fig 4A). Importantly, “rescuing” hTERT expression in the presence of miR-532/miR-3064 mimics enhances the proliferation and invasion of ES-2 cells (Fig 4B). On the other hand, the silencing of hTERT expression by specific siRNA oligonucleotides in the presence of miR-532/miR-3064 inhibitors could suppress the proliferation and invasion of SKOV-3 cells (Fig 4A and 4B). To further confirm the role of hTERT in the function of miR-532/miR-3064 in OC cells, we examined the protein expression of Slug and Bax in OC cells transfected with miR-532/miR-3064 mimics (or miR-532/miR-3064 inhibitors) followed by hTERT overexpression (or hTERT knockdown). Interestingly, hTERT overexpression in ES-2 cells could increase the expression of Slug reduced by miR-532/miR-3064 mimic, and can also decrease the expression of Bax elevated by miR-532/miR-3064 mimic (Fig 4C, left panel). Reversely, hTERT knockdown in SKOV-3 cells suppressed miR-532/miR-3064 inhibitors-induced Slug expression and increased the expression of Bax reduced by miR-532/miR-3064 inhibitors (Fig 4C, right panel). Collectively, these results support that hTERT inhibition is a key contributor of miR-532/miR-3064's tumor suppressive roles in OC cells.

### miR-532 and miR-3064 inhibits OC growth in a mouse model

**Fig 5:**
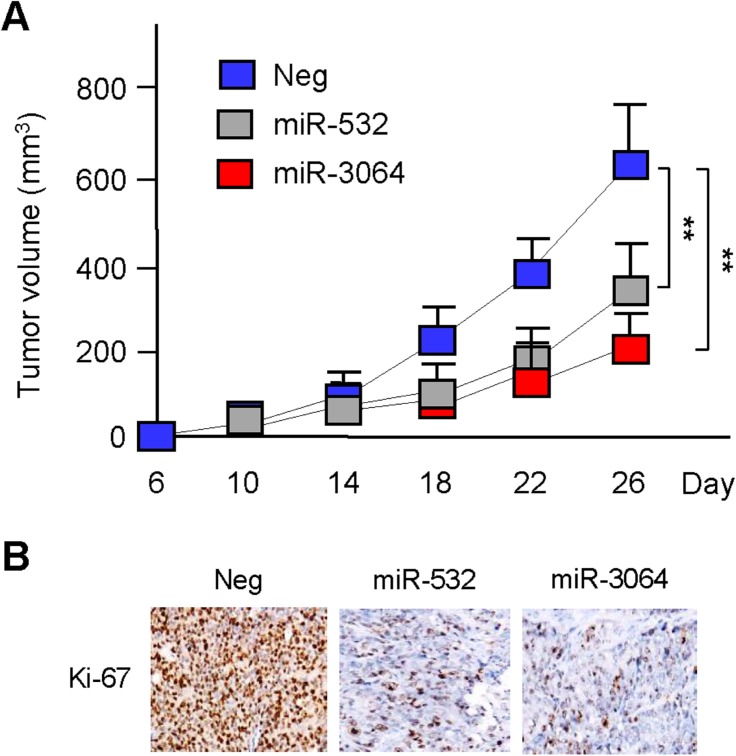
miR-532 and miR-3064 inhibit OC growth *in vivo*. (**A**) Nude mice were subcutaneously injected with 1.0 × 106 ES-2 cells stably overexpressing miR-532, miR-3064 or the corresponding negative control (Neg). After implantation for 6 days, tumor volume measurement began and was performed every 4 days (n = 8); (**B**) The xenograft tumors removed from nude mice were analyzed by immunohistochemistry for expression of Ki-67. **P < 0.01.

To evaluate the effects of miR-532 and miR-3064 on tumor growth in vivo, we manipulated the expression levels of miR-532 and miR-3064 in ES-2 cells, and then injected ES-2 cells into the flanks of nude mice to establish subcutaneous OC xenografts. ES-2 cells overexpressing miR-532 or miR-3064 exhibited attenuated tumorigenic ability 26 days after implantation (Fig 5A). Consistently, overexpression of miR-532 and miR-3064 downregulated the expression of Ki-67, a cell proliferation marker (Fig 5B). These data suggest that increasing miR-532 and miR-3064 levels can suppress the growth of OC cells *in vivo*.

## Discussion

The up-regulation of hTERT in various tumor types has been shown to be critical for the acquisition and maintenance of the metastatic ability of tumor cells, through promoting tumor growth and angiogenesis and by stimulating EMT, invasiveness and stemness of tumor cells [4–9, 22]. Although altered expression of several miRNAs has been involved in the regulation of hTERT [12–15], the epigenetic mechanisms that caused elevated hTERT expression in OC remain poorly characterized. In this study, we have attempted to identify tumor suppressor miRNAs that modulate hTERT expression in OC cells. Our *in vivo* experiments revealed that reduced levels of miR-532/miR-3064 correlate with hTERT up-regulation in OC tissues, and associate with worse OC patient survival, indicating that these two miRNAs could be causal factors in the overexpression of hTERT in OC. Our *in vitro* results further show that the direct inhibition of hTERT achieved by transient transfection of miR-532/miR-3064 mimics can repress OC cell proliferation and invasion. In an OC xenograft model, overexpressing miR-532/miR-3064 significantly decreased tumor growth of OC cells *in vivo*. Therefore, the use of these two miRNAs to target hTERT might provide an exciting avenue for the development of new cancer treatments.

Both genetic and epigenetic mechanisms play a role in hTERT-induced EMT and tumor metastasis [21, 23, 24, 25]. hTERT was shown to stimulate the EMT program through the Wnt/β-catenin pathway [21]. hTERT can also act as a coactivator of c-Myc to induce the expression of heparanase, which in turn enhances the invasion and metastasis of gastric cancer cells [23]. Recent studies demonstrated that hTERT and ZEB1 form a complex, which directly regulates E-cadherin to promote EMT in colorectal cancer cells [24]. We observed that hTERT expression was positively associated with Slug expression in OC cells, suggesting that Slug is the key mediator of hTERT-dependent OC aggressiveness. This finding is consistent with an early report that Slug is required for hTERT-induced EMT and invasion in OC cells [5]. Interestingly, hTERT enhances the growth, migration and invasion of gastric cancer cells via the indirect upregulation of ITGB1, through the repression of tumor suppressor miR-29a [25]. Therefore, one possible explanation of our observations is that hTERT-induced repression of miRNAs might contribute to the upregulation of Slug in OC cells.

Previous studies have reported that higher miR-532 expression correlates with longer survival of OC patients [26]. However, our knowledge of the molecular mechanisms mediating miR-532's function in OC specifically, has been limited. Here, we provided new evidence that the tumor suppressive roles of miR-532 in OC cells is mediated, at least in part, by the down-regulation of hTERT oncogene. It is well established that one single miRNA could target multiple mRNAs, the other miR-532 targets in OC cells require further investigation.

Until now, little has been known about the role of miR-3064 in human tumors. We report for the first time here that miR-3064 controls the expression of hTERT, and verified its tumor suppressor functions in the suppression of OC cell proliferation and invasion. Consistent with our findings, miR-3064 was shown to be down-regulated in colon cancer tissue relative to normal tissue [27]. Thus, our data indicated that this miRNA might exert a common anti-tumor effect in various cancers.

## Conclusions

In conclusion, our data suggest that hTERT overexpression might be caused by the loss of miR-532/miR-3064 (two direct suppressors of hTERT), and has a key role in enhancing OC cell proliferation and invasion. Re-expression of miR-532 or miR-3064 might be a possible strategy for the treatment of OC.

## Supporting information

S1 Table Association between miR-532/miR-3064 expression and clinicopathological characteristics of epithelial ovarian cancer. (DOCX)
