# Supplementary material for: MicroRNA-532 and microRNA-3064 inhibit cell proliferation and invasion by acting as direct regulators of human telomerase reverse transcriptase in ovarian cancer
Source: PLoS One. 2017 Mar 14;12(3):e0173912. doi: 10.1371/journal.pone.0173912 (PMC5349679; doi:10.1371/journal.pone.0173912)
Supplement: S1 Table — (DOCX) [file pone.0173912.s001.docx]

**Table S1. Association between miR-532/miR-3064 expression and clinicopathological characteristics of epithelial ovarian cancer.**

| Characteristics | Cases no. | miR-532 expression | *P* | miR-3064 expression | *P* |
| --- | --- | --- | --- | --- | --- |
|  |  | Low (n, %) |  | Low (n, %) |  |
| Age (year) |  |  |  |  |  |
| <50 | 28 | 15 (53.6) | NS | 14 (50.0) | NS |
| ≥50 | 32 | 16 (50.0) |  | 15 (46.9) |  |
| FIGO stage |  |  |  |  |  |
| I~II | 27 | 8 (29.6) | 0.0001 | 9 (33.3) | 0.0038 |
| III~IV | 33 | 27 (81.8) |  | 24 (72.7) |  |
| Histologic grade |  |  |  |  |  |
| Grade 1 | 13 | 4 (30.8) | 0.034 | 3 (23.0) | 0.032 |
| Grade 2 | 15 | 6 (40.0) |  | 7 (46.7) |  |
| Grade 3 | 32 | 22 (68.8) |  | 21 (65.6) |  |
| Residual tumor size |  |  |  |  |  |
| <1 cm | 40 | 21 (52.5) | NS | 20 (50.0) | NS |
| ≥1 cm | 20 | 10 (50.0) |  | 11 (55.0) |  |
| Lymph node Metastasis |  |  |  |  |  |
| Yes | 10 | 9 (90.0) | 0.011 | 9 (90.0) | 0.015 |
| No | 50 | 20 (40.0) |  | 21 (42.0) |  |

NS: not significant.
